# The lytic activity of VSV-GP treatment dominates the therapeutic effects in a syngeneic model of lung cancer

**DOI:** 10.1038/s41416-019-0574-7

**Published:** 2019-09-18

**Authors:** Liesa-Marie Schreiber, Carles Urbiola, Krishna Das, Bart Spiesschaert, Janine Kimpel, Fabian Heinemann, Birgit Stierstorfer, Philipp Müller, Monika Petersson, Patrik Erlmann, Dorothee von Laer, Guido Wollmann

**Affiliations:** 1 Division of Virology, Medical University of Innsbruck, Innsbruck, Austria; 2 Christian Doppler Laboratory for Viral Immunotherapy of Cancer, Innsbruck, Austria; 3 ViraTherapeutics GmbH, Innsbruck, Austria; 4 Boehringer Ingelheim Pharma GmbH & Co. KG, Biberach a.d. Riss, Germany

**Keywords:** Cancer immunotherapy, Cancer immunotherapy

## Abstract

**Background:**

Oncolytic virotherapy is thought to result in direct virus-induced lytic tumour killing and simultaneous activation of innate and tumour-specific adaptive immune responses. Using a chimeric vesicular stomatitis virus variant VSV-GP, we addressed the direct oncolytic effects and the role of anti-tumour immune induction in the syngeneic mouse lung cancer model LLC1.

**Methods:**

To study a tumour system with limited antiviral effects, we generated interferon receptor-deficient cells (LLC1-IFNAR1^−/−^). Therapeutic efficacy of VSV-GP was assessed in vivo in syngeneic C57BL/6 and athymic nude mice bearing subcutaneous tumours. VSV-GP treatment effects were analysed using bioluminescent imaging (BLI), immunohistochemistry, ELISpot, flow cytometry, multiplex ELISA and Nanostring® assays.

**Results:**

Interferon insensitivity correlated with VSV-GP replication and therapeutic outcome. BLI revealed tumour-to-tumour spread of viral progeny in bilateral tumours. Histological and gene expression analysis confirmed widespread and rapid infection and cell killing within the tumour with activation of innate and adaptive immune-response markers. However, treatment outcome was increased in the absence of CD8^+^ T cells and surviving mice showed little protection from tumour re-challenge, indicating limited therapeutic contribution by the activated immune system.

**Conclusion:**

These studies present a case for a predominantly lytic treatment effect of VSV-GP in a syngeneic mouse lung cancer model.

## Background

The development of oncolytic virotherapy has gained significant momentum in recent years with the clinical approval of the first oncolytic virus (Talimogene laherparepvec; Imlygic™) in the western hemisphere^1^ and its enhanced therapeutic efficacy when combined with established immunotherapies.^2,3^ Oncolytic virotherapy exploits direct and indirect mechanisms to attack malignancies. There is an initial tumour-selective viral replication and tumour cell killing, which subsequently leads to activation of innate and adaptive immunity. The activation of anti-tumour immunity holds the potential to induce long-lasting tumour remissions and immunological memory formation.^4–6^

Variants of vesicular stomatitis virus (VSV) have been thoroughly investigated for their oncolytic potential.^7–9^ VSV belongs to the family of *Rhabdoviridae*^10^ and its rapid replication cycle and wide host cell range make for a promising therapeutic agent.^11^ Tumour selectivity of VSV is predominantly based on defects in the antiviral defence capabilities of malignant cells,^8^ a feature commonly seen in many human malignancies.^12,13^ VSV-GP is a chimeric VSV variant with its glycoprotein (G) replaced by the lymphocytic choriomeningitis virus (LCMV) derived glycoprotein (GP). This results in the abrogation of VSV’s neurotoxicity without sacrificing its oncolytic potential as shown in a variety of different preclinical tumour models.^14–17^ In addition, pre-existing immunity is absent in the general population and induction of a neutralising antibody response is reduced,^18^ making systemic delivery possible. The clinical proof-of-concept of successful targeting disseminated lesions after intravenous injection was recently shown with another oncolytic virus.^19^

The overall therapeutic effect of virotherapy is shaped by the interaction between virus, tumour cells and the immune system.^20,21^ In immuno-compromised hosts with human tumour xenografts the treatment outcome is directly linked to the lytic activity within the tumour with only innate immune components responding to the viral challenge.^22^ Conversely, immune-competent models are paramount to assess the immune response in both trajectories—the potential anti-tumour immune activation as well as its virus-countering effects.^20,23^ VSV-based virotherapy has been preclinically tested on numerous xenograft and syngeneic models,^24^ with the latter displaying an effective induction of anti-tumour immunity in a number of VSV-treated cancer models.^25–27^ So far, most of these studies in immune-competent hosts point towards a significant immune-response contribution to the overall therapeutic effect of VSV treatment.

For this study we generated a mouse Lewis lung cancer LLC1 made highly permissive for VSV-GP via type 1 interferon receptor knockout. We could demonstrate that resistance to type 1 IFN-mediated antiviral protection in vitro translates to an increased and prolonged intratumoural virus activity in vivo, which resulted in complete remission of established subcutaneous tumours both in immune-competent and -deficient hosts. However, despite significant activation of innate and adaptive immune responses by VSV-GP, their contribution to the overall therapeutic effect was ineffectual in this particular tumour setting. We believe this model can inform on studies of potentially rare clinical instances in which a tumour response to virotherapy is lytic-dominant with little contribution of anti-tumour immunity.

## Methods

### Cell lines and viruses

LLC1 cells were obtained from ATCC (American Type Culture Collection, Manassas, VA, USA; #CRL-1642) and maintained in high glucose DMEM (Lonza, Basel, Switzerland) supplemented with 10% heat inactivated FCS, 4mM L-Glutamine (Gibco, Carlsbad, California, USA), 100 units/ml penicillin and 0.1 mg/ml streptomycin (Gibco) at 37 °C/5% CO_2_. VSV-GP, VSV-GP-GFP, VSV-GP-Luciferase and VSV-GP-ΔG have been described previously.^14,17^ Viruses were propagated and titred on BHK-21 cells (ATCC).

### Generation of IFNAR1^−/−^ LLC1 cells

Plasmids encoding Transcription Activator-Like Effector Nucleases (TALEN) sequences targeting the murine heterodimeric interferon type I receptor complex *Ifnar1* gene (NM_010508.2) were purchased from GeneCopoeia (Maryland, USA). The following pairwise target sites were chosen: (1) L: TCCTGAGAATATAGACGTC – R: TGCTCCACTTTAGGGTGTA; (2) L: TGCCTGAATGTCAACATAC – R: TGTGTCCAGTAAAGAGAAT; (3) L: TCTTCGTGGAATGAGGTTG – R: TGGCGGCTTCTTACCTGTG. LLC1 cells were transfected with three TALEN pairs (0.6 µg each) using Trans-IT (Mirus, Madison, Wisconsin, USA) following manufacturer’s instructions.

### Flow cytometry and single cell sorting

Cell suspensions were stained for flow cytometry analysis using the following specific antibodies: anti-IFNAR1 mouse (1:250, MAR1–5A3, BioLegend, San Diego, California, USA); APC-conjugated anti-mouse-IgG1 from goat (1:100, Jackson ImmunoReasearch, Suffolk, UK) and sorted into single cell clones using FACS Aria (BD Biosciences, Schwechat, Austria). For quantification of cell surface IFNAR1, selected clones were incubated using the same antibodies and analysed using FACS Canto II (BD Biosciences).

### Microscopic analysis

5 × 10^4^ cells/well were seeded in 24-well plates and treated with 500 U/mL of universal IFN-α A/D (PBL, Piscataway, New Jersey, USA) for 16 h or left untreated. Cultures were infected with VSV-GP-GFP at MOI 0.1 for 24 h before assessing fluorescence expression.

### Cell viability and IFN-I resistance assay

2 × 10^4^ cells/well were plated in 96-well plates and treated with universal IFN-α A/D (PBL) at different concentrations for 16 h prior virus infection with various VSV-GP concentrations. Seventy-two hours later MTT viability assay was performed as described previously.^17^

### In vivo studies

The studies were performed in compliance with the Austrian experimentation law (animal trial permission granted by the Federal Ministry of Science, Research and Economy BMWFW-66.011/0012-WF/V/3b/2016 and BMWFW-66.011/0041-WF/V/3b/2016). Six to eight-week-old female athymic Rj:NMRI-Foxn^1nu/nu^ mice or C57BL/6JRj mice weighing 16–20 g were obtained from Janvier (Le Genest St Isle, France). A health status certificate was supplied with every mouse delivery. Tumours were implanted by subcutaneous injection of 100 µl of 1 × 10^6^ LLC1-IFNAR1^−/−^ or 5 × 10^5^ LLC1 cells in the flank of syngeneic C57BL/6JRj or athymic Rj:NMRI-Foxn^1nu/nu^ mice. Tumour size was measured twice a week with a calliper and volume was calculated using the formula: length × width² × 0.4. Treatment commenced when tumours reached a size of 0.05 to 0.07 cm^3^. PBS-based solutions containing 10^8^ TCID_50_ of virus were used for intratumoural (30 µl) or intravenous (100 µl) injection. Mice were sacrificed when tumour size reached 0.8 cm³ or tumours showed signs of ulcerations. Animals were euthanised by CO_2_ asphyxiation and cervical dislocation or via short-term isoflurane anaesthesia followed by cervical dislocation. For bilateral tumours 3 × 10^5^ LLC1-IFNAR1^−/−^ cells were injected into the flanks of C57BL/6JRj and Rj:NMRI-Foxn^1nu/nu^ mice. For luciferase imaging, Lumina system was used (IVIS Lumina II, Perkin Elmer, Waltham, Massachusetts, USA) as described in ref. ^16^ For CD8^+^ T cell depletion, mice were intraperitoneally injected with 100 µg anti-mouse CD8 (clone YTS 169.4, Hölzel Diagnostika GmbH, Köln, Germany) or anti-IgG2b-a-kIH (clone LTF-2, Hölzel Diagnostika GmbH) antibody. Depletion was repeated on days 0, 2, 6 and 10 post virus treatment. Mice were treated intravenously with 10^8^ TCID_50_ VSV-GP on days 0, 4 and 8. CD8^+^ T cells depletion was confirmed by staining against CD3 (PE-Cy7, Clone 17A2, 1:200, BD Biosciences), CD8 (Pacific Blue, Clone 53–6.7, 1:750, BD Biosciences) and CD43 (FITC, Clone 1B11, 1:100, BioLegend). The studies were designed in compliance with ARRIVE guidelines. Detailed additional information is provided in supplementary methods (Methods S1).

### Isolation of splenocytes and tumour infiltrating immune cells

Splenocytes were isolated via 40 µm cell strainer. Erythrocytes were lysed, and PBS-washed cells were resuspended in appropriate buffer for flow cytometry or IFNγ ELISpot. LLC1-IFNAR1^−^/^−^ tumours were processed using the mouse tumour dissociation kit (Miltenyi Biotec Bergisch Gladbach, Germany) and the GentleMACS dissociator (Miltenyi Biotec) according to manufacturer’s instructions. Single cell suspension was filtered through 70 µm cell strainer, washed with medium and centrifuged for 10 min at 1600rpm. Approximately 5 × 10^7^ cells were layered on top of Lympholyte-M solution (Cedarlane Burlington, Ontario, Canada). After density gradient centrifugation, cells were removed from the interphase and washed using PBS. FC receptors were blocked by incubating cells for 20 min at 4 °C with FACS buffer containing FCR block CD16/32 (BD Pharmingen) and rat and hamster serum (Jackson ImmunoResearch, Ely, United Kingdom). Cells were subsequently stained for flow cytometry as described below.

### Detection of VSV-GP specific CD8^+^ T cells by flow cytometry

Cells were stained with the H-2Kb VSV-NP-PE-tetramer (Biomedica) followed by staining for surface markers using the following antibodies: CD45.2-PerCP-Cy5.5 (Clone 104, BioLegend), CD90.2-AF488 (Clone 30-H12, BioLegend), CD8-BV510 (Clone 53–6.7, BioLegend), CD14-APC-Cy7 (Clone Sa14–2, BioLegend), CD19-APC-Cy7 (Clone 6D5, BioLegend) and CD4-APC-Cy7 (Clone GK1.5, BioLegend). Non-viable cells were stained using LIVE/DEAD™ Fixable Near-IR Dead Cell Stain Kit (Thermofisher). Samples were analysed using FACS Canto II (BD Biosciences) and data analysis was performed using the FlowJo software (FlowJo LLC, Oregon, USA).

### IFNγ ELISpot

IFNγ secretion of splenocytes was investigated using the IFNγ ELISpotPLUS kit (MabTech Nacka Strand, Sweden). Briefly, 2.5 × 10^6^ splenocytes were incubated overnight at 37 °C with 5 × 10^4^ LLC1-IFNAR1^−/−^ cells or 10 µg/mL mSurvivin peptides (mSur_20–28_: ATFKNWPFL, mSur_57–64_: CFFCFKEL or mSur_97–104_: TVSEFLKL, gift from R. Amann) or VSV-NP peptide (VSV-NP_52–59_: RGYVYQGL, GeneScript Piscataway, NJ, USA) in IFNγ capture antibody pre-coated plates. Development of spots was performed as advised by the manufacturer and enumerated using the ImmunoSpot S6 reader (CTL, Bonn, Germany).

### Immunohistochemical analysis

Resected tumours were cut in two halves; one was stored in RNAlater (Thermo Fisher Scientific, Waltham, MA USA) at 4 °C for NanoString analysis (see below), one was fixed in 4% paraformaldehyde prior to paraffin embedding (FFPE). Three-micron tissue sections were de-waxed with xylene, rehydrated in a graded ethanol series and blocked with 3% hydrogen peroxide. Antigen retrieval was performed for all primary antibodies except VSV-N (no pre-treatment) by heating the sections in Tris-EDTA buffer (95 °C; pH 9.0) for 20 min. Sections were incubated for 1 h at room temperature with the following primary antibodies: VSV-N (1:250; Kerafast, Boston, MA, USA; #EB0009), cleaved Caspase 3 (1:2500; Cell signalling technology, Frankfurt, Germany; #9667), CD8a (1:100; eBioscience, Waltham, MA USA; #14–0808), CD4 (1:200, eBioscience; #14–9766) or PD-L1 (1:100, R&D Systems, Minneapolis, MN, USA; #AF1019). A biotinylated or HRP-conjugated antibody followed by DAB or Refine Red chromogen was used for detection. Haematoxylin (Bond™ Polymer Refine Detection, #37072; Leica Biosystems) was applied for counterstaining. Staining was performed on the automated Leica IHC Bond-III™ platform (Leica Biosystems). Microscopy was conducted with a Zeiss AxioImager M2 microscope (Zeiss, Oberkochen, Germany), slide scans were obtained using a Zeiss AxioScan Z1 scanner. Density of IHC stain positive cells was quantified with the image analysis software Halo 2.1 (IndicaLab, Corrales, New Mexico, USA) using the Cytonuclear IHC analysis module.

### NanoString analysis

Tumours were homogenised with the SpeedMill PLUS (Analytik Jena, Jena, Germany) and RNA was extracted using Phenol:Chloroform:Isoamyl Alcohol (25:24:1) (Sigma–Aldrich, USA) and MagMAX-96 Total RNA Isolation Kit (Thermo Fisher) following manufacturer’s instructions. Extracted RNA was analysed for differential expression by means of the nCounter PanCancer Immune Profiling Panel and the nCounter FLEX Analysis System (NanoString Technologies, Seattle, WA, USA). Profiled data were pre-processed following the manufacturer’s recommendations.^28,29^ Heatmaps of NanoString data were generated using TreeView.^30^

### Intratumoural cytokine levels

Resected tumours were snap frozen and homogenised using the SpeedMill PLUS (Analytik Jena) in 500 µl Procartaplex cell lysis buffer (Thermo Fisher) per 100 mg tissue. Homogenate was centrifuged at 6000 × *g* for 20 min. Lysates were analysed using the Procartaplex Cytokine & Chemokine 26-Plex Mouse Panel 1 on a Luminex MAGPIX fluorescence imager (Thermo Fisher Scientific) and LEGENDplex^TM^ Mouse Anti-Virus Response Panel (13-plex) (BioLegend) followed by flow cytometry analysis. Cytokine concentrations were normalised to total protein concentration in the lysate measured using the Pierce BCA protein assay (Thermo Fisher Scientific).

### Statistical analysis

GraphPad Prism software (Version 7, GraphPad Software, La Jolla, California, USA) was used for statistical analysis. ANOVA test was applied to assess significance levels. Kaplan–Meier survival curves were compared using the Log-rank (Mantel-Cox) test. Data are presented as mean ± SEM or SD as noted. Statistically significant differences were encoded as follows: *p < 0.05; **p < 0.01; ***p < 0.001; ****p < 0.0001.

## Results

### Interferon sensitivity limits VSV-GP activity on murine lung cancer cell line LLC1 in vitro

**Fig. 1:**
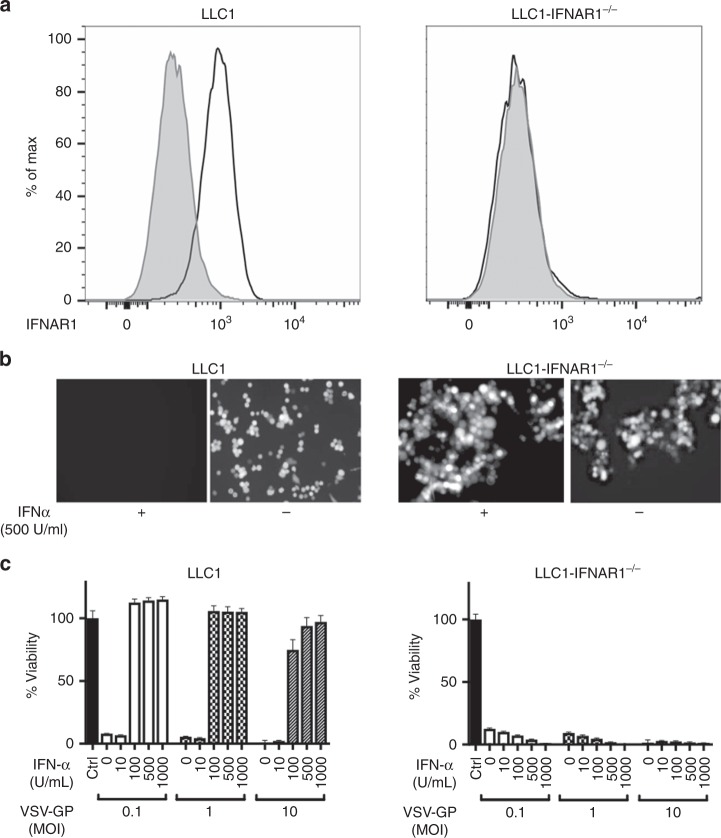
Interferon responsiveness limits VSV-GP activity on murine lung cancer cell line LLC1 in vitro. **a** Surface expression of IFNAR1 on LLC1 wt and IFNAR1^−/−^ was analysed by flow cytometry using mIFNAR1 specific antibody with only secondary antibody (grey) as control. **b** For microscopic analysis of VSV-GP-GFP infection in LLC1 wildtype and IFNAR1^−/−^, cells were pre-incubated with 500 U/mL universal IFN-α overnight or left untreated and subsequently infected with VSV-GP-GFP at an MOI 0.1 for 24 h. **c** Survival of LLC1 wt and IFNAR1^−/−^ cells upon VSV-GP infection at increasing dose (MOI 0.1,1,10) and IFN-α pre-treatment (0–1000 U/mL overnight) was measured 72 h post infection using MTT assay. Data are shown as mean ± SEM (*n* = 4)

While various aberrations in the type I interferon signalling pathway are described for many human tumour cell lines,^31,32^ interferon insensitivity is often lacking in murine models of cancer.^14,17,33^ To generate a tumour system with limited antiviral effect of type I interferon signalling that more closely mimics the condition found in many human cancers, the subunit 1 of the heterodimeric interferon type I receptor complex (IFNAR1) was knocked-out in murine LLC1 lung cancer cells. Consequently, surface expression of IFNAR1 was absent in LLC1-IFNAR1^−/−^ cells compared to parental LLC1 cells (Fig. 1a). In contrast to LLC1 cells, expression of the virally encoded reporter gene GFP was not affected by pre-incubation with IFN-α in LLC1-IFNAR1^-/-^ cells (Fig. 1b). We next assessed the outcome of VSV-GP infection on LLC1 cells after pre-incubation with various IFN-α concentrations using an MTT-based viability assay. Parental LLC1 cells showed near complete protection from VSV-GP infection in contrast to IFNAR1-deficient LLC1 cells that were highly sensitive to VSV-GP infection and killing (Fig. 1c).

### The in vivo efficacy of VSV-GP in the syngeneic LLC1 lung cancer model is dose dependent and correlates with tumour interferon sensitivity

**Fig. 2:**
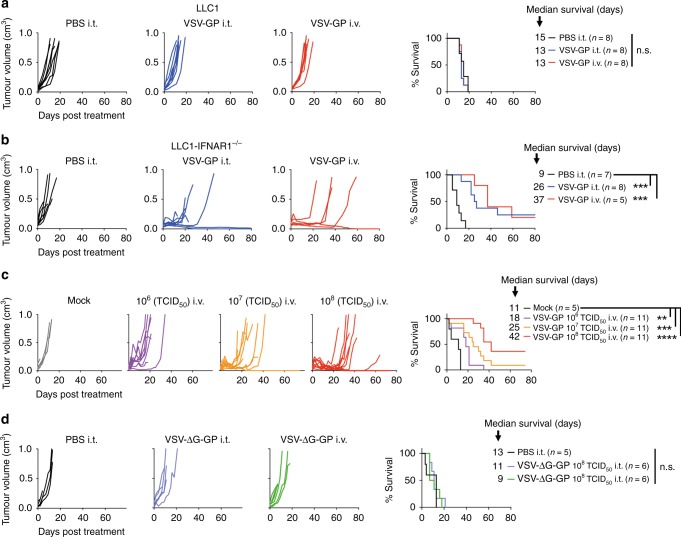
The in vivo efficacy of VSV-GP in the syngeneic LLC1 lung cancer model is dose dependent and correlates with tumour interferon sensitivity. Tumours were implanted in C57BL/6J mice by subcutaneously injecting 5 × 10^5^ LLC1 wt (**a**) or 1 × 10^6^ IFNAR1^-/-^ cells (**b**) into the right flank. Intratumoural or intravenous treatment with VSV-GP at a dose of 10^8^ TCID_50_ was initiated when tumours reached a size of 0.05–0.07 cm^3^. Established LLC1-IFNAR1^-/-^ tumours were treated intravenously with a single dose of 10^6^, 10^7^ or 10^8^ TCID_50_ VSV-GP (**c**) or 10^8^ TCID_50_ replication-deficient VSV-ΔG-GP via intratumoural or systemic route (**d**). Individual tumour volume graphs and Kaplan–Meier survival curves are shown (*p < 0.05; **p < 0.01; ***p < 0.001; ****p < 0.0001)

Efficacy of VSV virotherapy in syngeneic mouse tumour models has been described both in tumours highly susceptible for VSV infection^34^ as well as in models with limited viral replication within the tumour tissue.^25^ To address whether the interferon response of LLC1 cells correlates with oncolytic VSV-GP efficacy in vivo, we compared the treatment outcome in subcutaneous LLC1 and LLC1-IFNAR1^−/−^ tumours in syngeneic C57BL/6J mice. Single intratumoural or systemic VSV-GP injections of 10^8^ TCID_50_ showed no treatment effect on LLC1 tumours (Fig. 2a). In contrast, both systemic and intratumoural virus treatment of IFNAR1^−/−^ tumours induced strong tumour remission (****p < 0.0001 between day 9–20) (Fig. 2b). Although most tumours started to relapse around day twenty to thirty, median survival was significantly (***p < 0.001) increased from 9 days post treatment in PBS-treated mice to 26 days in VSV-GP i.t. and 37 days in VSV-GP i.v. treated mice. The VSV-GP treatment effect showed a dose dependency in both time-to-progression as well as in overall survival (Fig. 2c). Compared to the highest virus dose (10^8^ TCID_50_), tumours treated with 10^7^ TCID_50_ VSV-GP relapsed earlier (day 10–20) while a low dose of 10^6^ TCID_50_ showed only a partial response and rapid relapse. Median survival increased from 18 days (10^6^ TCID_50_), to 25 (10^7^ TCID_50_) and 42 days (10^8^ TCID_50_ days), respectively, compared to control at 11 days. To address if virus replication is required for the therapeutic effect we treated LLC1-IFNAR1^−/−^ tumours with a replication-deficient VSV-GP variant (VSV-ΔG-GP) via intratumoural or systemic application. These tumours were completely resistant to replication-incompetent VSV-ΔG-GP therapy with no objective response or survival benefit compared to control (Fig. 2d).

### Therapeutic effect of VSV-GP on LLC1-IFNAR1^−/−^ tumours is independent of anti-tumour immune activation

We next addressed whether the adaptive immune status of the host affects the outcome of the VSV-GP treatment in LLC1 or LLC1-IFNAR1^−/−^ tumours. As in immune-competent hosts, subcutaneous LLC1 tumours grown in athymic NMRI-nu mice were resistant to intratumoural VSV-GP treatment (Fig. S1A). In contrast, LLC1-IFNAR1^−/−^ tumours showed complete and lasting remission after VSV-GP treatment (Fig. S1B). This suggests that the adaptive immune system is a negligible factor for lack of a therapeutic effect of VSV-GP in parental LLC1 tumours.

**Fig. 3:**
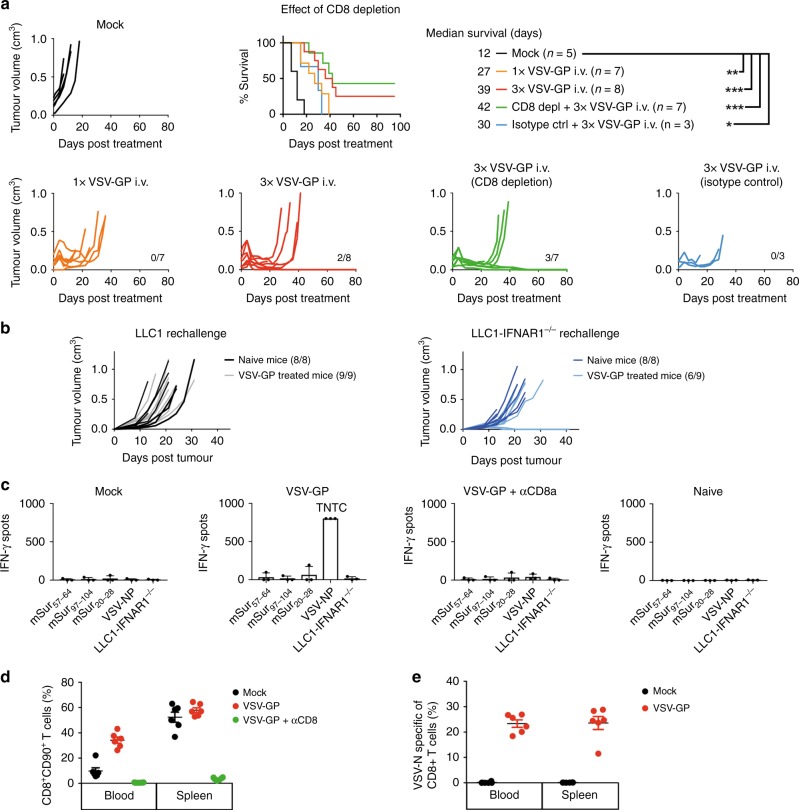
VSV-GP treatment effect on LLC1-IFNAR1^−/−^ tumours is independent of adaptive immune activation. **a** To selectively address the contribution of cytotoxic T cells to VSV-GP treatment, C57BL/6J mice bearing LLC1-IFNAR1^−/−^ tumours were depleted for CD8^+^ T cells using a monoclonal antibody at days -2, 0, 2, 6 and 10 respective to single systemic virus (10^8^ TCID_50_) treatment. Individual tumour volume graphs and Kaplan–Meier survival curve are shown (*p < 0.05; **p < 0.01; ***p < 0.001). **b** Mice from two separate experiments that showed LLC1-IFNAR1^−/−^ tumour long-term remission after VSV-GP treatment were re-challenged subcutaneously with 1 × 10^6^ of either parental or IFNAR1^−/−^ LLC1 cells into the left flank and monitored for tumour outgrowth. **c** Reactivity of splenocytes isolated from mock, VSV-GP or VSV-GP + CD8a depletion—treated or completely naïve mice against three different survivin epitopes, VSV-NP peptide as well as LLC1-IFNAR1^−/−^ tumour cells are depicted as number of IFNγ spots per 2.5 × 10^6^ cells. Numbers of spots of three animals per treatment group are presented as mean of three technical replicates, after subtraction of background signal (medium only). TNTC = spots too numerous to count. **d** Flow cytometry was used to quantify overall and VSV-GP specific CD8^+^ T cell response in whole blood and spleen on days 6 and 7 post virus treatment, respectively. After gating out non-viable cells, monocytes, myeloid cells and B cells percentages of CD8^+^CD90^+^ T cells among CD45^+^ leukocytes are shown. **e** Frequencies of VSV-GP specific CD8^+^ T cells labelled by VSV-NP tetramer are shown from blood and spleen samples

To test whether a CD8^+^ T cell mediated anti-tumour component contributes to the overall therapeutic effect of VSV-GP in the permissive LLC1-IFNAR1^−/−^ tumour, CD8^+^ T cells were depleted using a monoclonal antibody. Depletion resulted in an almost complete elimination (>95%) of the CD8^+^ cells (Fig. S2). Intravenous treatment with a single or triple injection (days 0, 4, 6) of 10^8^ TCID_50_ VSV-GP resulted in 100% remission (Fig. 3a). Triple treatment showed a trend for survival benefit compared to single dose therapy (39 vs. 27 days median survival; n.s.). Importantly, depletion of CD8^+^ T cells did not result in reduced efficacy (overall survival 3/7 compared to 2/7 in the non-depleted treatment group; n.s.).

To assess a potential memory response for long-term tumour control, mice cured from LLC1-IFNAR1^−/−^ tumours by VSV-GP therapy (survival >80 days) were re-challenged via contralateral subcutaneous injection of parental or IFNAR1-deficient LLC1 cells and monitored for tumour outgrowth. Naïve, age-matched animals developed LLC1 wt or IFNAR1^−/−^ tumours within 8 days. In LLC1-IFNAR1^−/−^ tumour-cured mice, challenge with parental LLC1 tumours resulted in tumour growth equal to naïve mice (Fig. 3b). For IFNAR1^−/−^ tumour challenge, grafting rate was 100% with 6/9 tumours growing with similar kinetics as in naïve mice (Fig. 3b), indicating only a partial and non-robust immunological anti-tumour memory response.

To assess the antiviral versus the antitumour immune response during active VSV-GP treatment, splenocytes from mice bearing LLC1-IFNAR1^−/−^ tumours were harvested 7 days after systemic virus treatment and analysed using an IFNγ ELISpot assay. LLC1 tumours highly express the tumour-associated antigen, survivin,^35^ which has been successfully targeted by various immunotherapeutic approaches.^36^ We confirmed survivin expression in LLC1-IFNAR1^−/−^ cells (Fig. S3) and hypothesised a potential induction of anti-tumour T cells due to VSV-GP therapy would include a population of survivin-specific T cells. However, stimulation of splenocytes with synthetic peptides representing previously described survivin epitopes^37,38^ or with LLC1-IFNAR1^−/−^ cells did not result in enhanced IFNγ secretion by T cells in any of the treatment groups indicating lack of high frequency of T cells reactive to survivin or other antigens expressed by LLC1-IFNAR1^−/−^ (Fig. 3c). As expected, a strong IFNγ response resulted from recognition of the immunodominant epitope of VSV-GP (VSV-NP_52–59_)^39^ by the splenocytes of VSV-GP treated mice, which was absent in mock treated or tumour-free naïve mice (Fig. 3c). Of note, VSV-GP can result in higher unspecific IFNγ secretion by splenocytes even in absence of peptide stimulation (data not shown)^40^ and therefore background activation was corrected. Specific IFNγ response was absent in splenocytes from VSV-GP treated mice when CD8^+^ T cells were depleted, suggesting that IFNγ secreting cells were indeed VSV-specific cytotoxic T lymphocytes (Fig. 3c, d). Flow Cytometry using peptide-MHC multimers confirmed approximately 23% of CD8^+^CD90^+^ cytotoxic T cells in blood and spleen were directed against the highly immunogenic virus epitope VSV-NP_52–59_ after single VSV-GP administration (Fig. 3e).

Together, these results suggest a predominantly lytic mode of action underlying the observed anti-tumour effects of VSV-GP in the LLC1-IFNAR1^−/−^ tumour model with only a weak long-term anti-tumour immunity in this specific tumour model.

### Intratumoural virus activity correlates with interferon resistance and CD8^+^ T cell deficiency

**Fig. 4:**
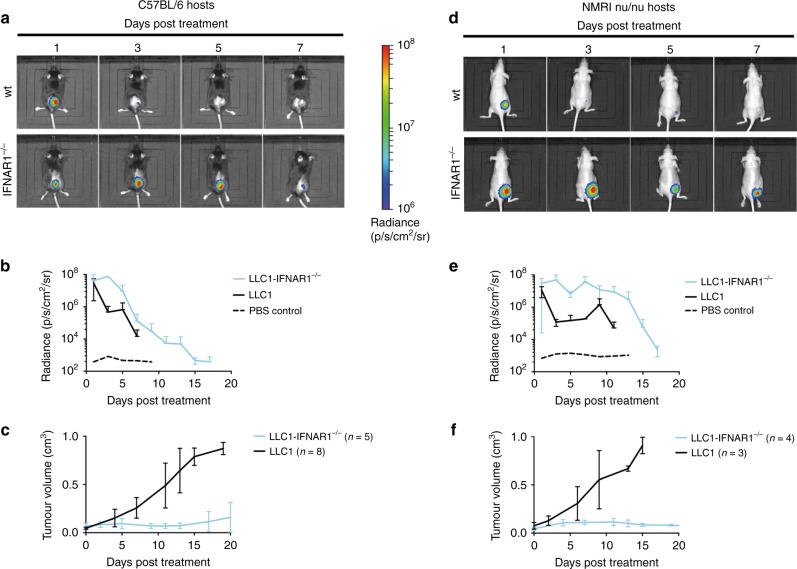
Interferon insensitivity and CD8^+^ T cell deficiency correlate with enhanced and prolonged intratumoural virus replication. Unilateral LLC1 wt or IFNAR1^−/−^ mouse lung tumours were grown in syngeneic C57BL/6 J mice (**a**–**c**) or athymic NMRI-nu/nu mice (**d**–**f**) and treated intratumourally with a single dose of 10^8^ TCID_50_ VSV-GP-Luciferase. Tumours were monitored every second day post treatment using the in vivo bioluminescence imaging (BLI) system IVIS. Representative BLI pictures of treated mice (**a**, **d**) and quantification of the average radiance in the tumour area (**b**, **e**) are shown (mean ± SD). Tumour growth after VSV-GP-Luciferase treatment is depicted with aligned time axis as mean ± SD in C57BL/6 J (**c**) and NMRI-nu/nu mice (**f**). Colour scale displays luminescence as photons/second/cm^2^/steradian (p/s/cm^2^/sr)

We next used a VSV-GP variant expressing firefly luciferase (VSV-GP-Luc) to monitor intratumoural virus activity depending on tumour permissiveness (IFNAR1 wt or deficient) and adaptive immune status (syngeneic C57BL/6J mice vs. athymic nude mice). In immune-competent syngeneic hosts, intratumoural injection of 10^8^ TCID_50_ VSV-GP-Luc resulted in comparable tumour-selective bioluminescence signals in parental LLC1 and LLC1-IFNAR1^−/−^ tumours at 24 h post infection, indicative of similar first-round activity independent of the IFNAR status in the tumour. However, little to no signal could be detected at later time points in parental LLC1, whereas the bioluminescence signal in VSV-GP-Luc treated LLC1-IFNAR1^−/−^ tumours increased over several days before starting to decline after 5 days (Fig. 4a, b) indicating active viral replication in interferon insensitive tumours (∆radiance IFNAR1^−/−^ vs wt LLC1 greater than 2 logs). The same dynamic pattern was observed in athymic nude mice (Fig. 4d). However, virus activity was significantly prolonged in VSV-GP-Luc treated LLC1-IFNAR1^−/−^ tumours (Fig. 4e) compared to the duration of virus replication in C57BL/6J mice, supporting the earlier findings of relapse-free long-term therapeutic outcome of VSV-GP treatment in immune-compromised hosts. In terms of therapeutic tumour control, VSV-GP-Luc was found to be as effective as VSV-GP on LLC1-IFNAR1^−/−^ tumours leading to rapid tumour remission in syngeneic C57BL/6J as well as NMRI-nu mouse model (Fig. 4c, f). We next addressed the question of potential tumour-to-tumour spread by monitoring VSV-GP-Luc mediated bioluminescence in a bilateral subcutaneous tumour setting. In immune-compromised NMRI-nu hosts, unilateral intratumoural VSV-GP-Luc injection resulted in virus spread to the contralateral site within 3 days (Fig. S4A). Remarkably, even in fully immunocompetent C57BL/6J mice unilateral VSV-GP-Luc injection generated a successful secondary virus infection in the contralateral tumour with a 7-day latency (Fig. S4B**)**.

### Widespread lytic activity of VSV-GP on LLC1-IFNAR1^−/−^ tumours is associated with T cell infiltration and immune activation

**Fig. 5:**
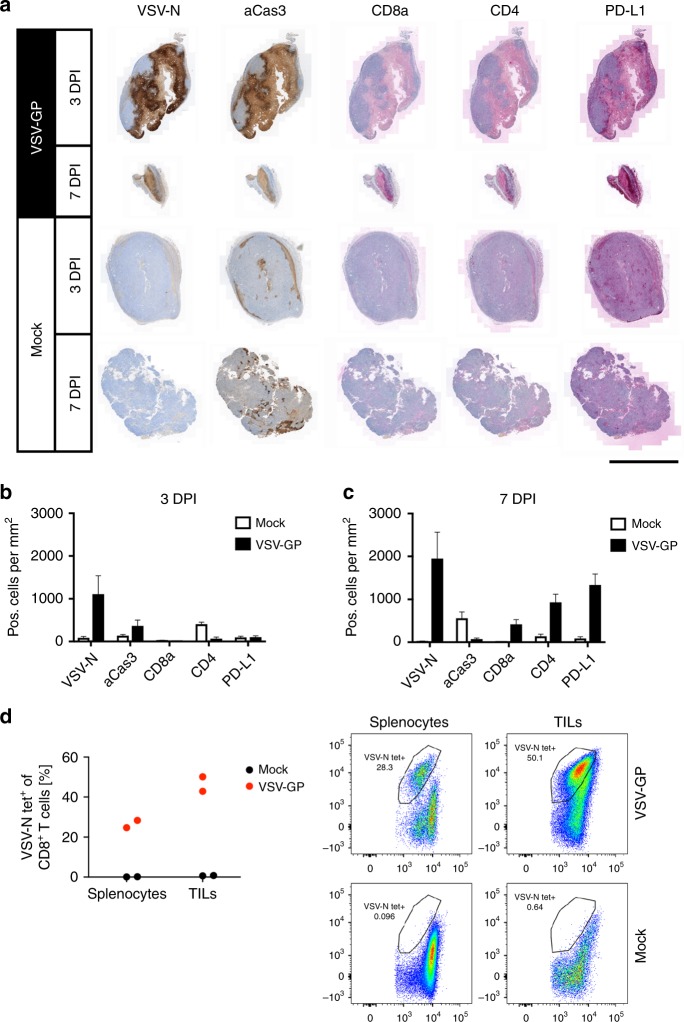
Lytic activity of VSV-GP on LLC1-IFNAR1^−/−^ tumours associated with T cell infiltration and immune activation. Established LLC1-IFNAR1^−/−^ tumours were treated with a single intravenous dose of 10^8^ TCID_50_ of VSV-GP and resected after 3 or 7 days, respectively. Immunostaining against VSV-N, activated caspase 3 (aCas3), CD8a, CD4 and PD-L1 was performed on fixed microsections. **a** The panel depicts representative pictures of a group size of six animals each. Scale bar 3 mm. The density of IHC positive cells in sections was analysed 3 days (**b**) and 7 days (**c**) post treatment and revealed significant immune activation at the latter time point. Data are shown as mean ± SEM (*n* = 6). **d** VSV-specific CD8^+^ T cells in spleen and tumour of the same animal were measured 7 days post treatment in flow cytometry using VSV-NP-MHC multimer. Frequencies of VSV-specific CD8^+^ T cell among CD90^+^CD8^+^ T cells are depicted in a graph (left panel) and as dot plots (right panel)

Finally, we were interested in the effect of VSV-GP treatment on the tumour microenvironment and host immune activation. LLC1-IFNAR1^−/−^ tumours were resected three and seven days post intravenous VSV-GP treatment. Immunohistochemical analysis confirmed extensive virus infiltration of and replication in tumour tissue and increased induction of apoptosis compared to mock treated tumours (Fig. 5a). The tumour-restricted virus infection induced a significant pan-tumour infiltration of CD8^+^ and CD4^+^ T cells in this otherwise non-T cell inflamed tumour model^41^ (Fig. 5b, c). This immune cell infiltration was associated with a strong upregulation of PD-L1 expression within the tumour microenvironment, increasing from 3 to 7 days following VSV-GP treatment. Flow cytometric analysis revealed that 7 days after VSV-GP application, 40–50% of CD8^+^ tumour infiltrating lymphocytes were specific for VSV (Fig. 5d, Fig. S5), almost twice the frequency detected in the spleen of the same animals suggesting virus specific T cells were enriched in the tumour microenvironment.

### Changes of intratumoural cytokine profile after VSV-GP treatment

**Fig. 6:**
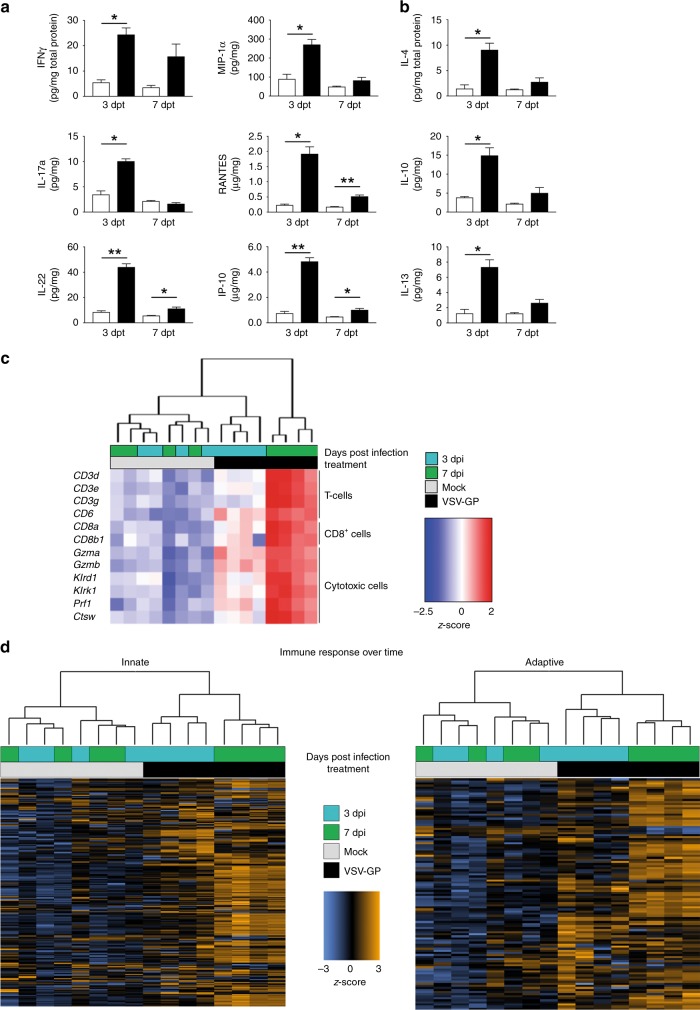
Proinflammatory cytokine release and activation of innate and adaptive immune responses after VSV-GP treatment. Established LLC1-IFNAR1^−/−^ tumours were treated with a single intravenous dose of 10^8^ TCID_50_ of VSV-GP and resected after 3 or 7 days, respectively. For quantification of cytokine levels, tumour lysates were assayed using Luminex multiplex technology to detect proinflammatory (**a**) and immune-suppressive cytokines (**b**). Data presented as mean ± SEM (*n* = 3) (*p < 0.05; **p < 0.01). RNA from tumour homogenates was used for transcriptome analysis via NanoString technology. A hierarchical cluster analysis (Euclidean distance; average linkage) for sample data were performed. The z-score-normalised gene expression intensity is presented with each column representing one individual tumour. Four tumours per group were used for analysis. Data shown are representative from two independent experiments. **c** Heatmap presents T cell signature genes from treated vs untreated tumours at 3 and 7 days post treatment. Genes were selected based on the cell type–annotation from the NanoString nCounter PanCancer Immune Profiling Panel gene list. Genes are ordered according to the cell type annotation. **d** Heatmaps visualising the differential expression of gene panels representative for innate and adaptive immune response, respectively

Systemic VSV-GP treatment resulted in significant intratumoural induction of proinflammatory factors IFN-γ, IL-17a, IL-22, MIP-1α (CCL3), RANTES (CCL5), and IP-10 (CXCL10) and anti-inflammatory cytokines IL-4, IL-10 and IL-13 3 and 7 days post treatment (Fig. 6a, b). Although not statistically significant, TNF-α, IL-12 and IL-18 were also increased at both time points (Fig. S6A). GM-CSF concentration in the tumour was not affected by VSV-GP treatment (data not shown). A separate experiment confirmed the dose- and replication-dependent effect of VSV-GP treatment on intratumoural cytokine levels 7 days post treatment (Fig. S6B). Replication-incompetent VSV-ΔG-GP treatment failed to elicit any cytokine response in tumour tissue.

### Transcriptome analysis of VSV-GP treated LLC1-IFNAR1^−/−^ tumours

Tumours were also processed for NanoString analysis to assess changes in the transcription of over 700 inflammatory, immune response and viral genes. Corroborating the histological analysis, the expression of immune cell type–specific genes, specifically of CD8^+^ cells, cytotoxic cells and T cells, was strongly upregulated over time after VSV-GP administration (Fig. 6c). This confirms the strong infiltration of T cells seen microscopically **(**Fig. 5a**)** and additionally suggests a pronounced immune cell activation. A more comprehensive analysis of the immune signature revealed a differentiated immune response over time in the treated tumours. Despite the knockout of the interferon receptor in these tumours, a strong innate immune response could be observed, increasing up to day 7 post infection **(**Fig. 6d, Table S1**)**. The adaptive immune signature also presented a differentiated upregulation that progressed at least up to day 7 post infection (Fig. 6d, Table S2). These data suggest that intratumoural VSV-GP activity results in a significant and broad upregulation of adaptive immune responses.

## Discussion

In the present study we generated and characterised a syngeneic mouse tumour model system that is highly permissive for oncolytic VSV-GP replication after local as well as systemic application. Although the tumour-specific infection and replication induced strong innate and adaptive immune responses, the therapeutic anti-tumour effect appears to be largely independent from the observed immune activation.

Many oncolytic viruses display a particular sensitivity towards innate antiviral immunity^42^ and utilise defects in these antiviral mechanisms to target cancer cells.^8,43,44^ Opposed to various human cancer models, which have been reported to commonly exhibit a reduced antiviral protection,^31,45,46^ most mouse tumour cell lines are sensitive to antiviral response mediators, such as type I IFNs.^14,17,33^ We therefore generated an IFNAR1 knockout mouse tumour cell line with disrupted IFN type 1 response. We chose to target IFNAR due to its upstream position in the innate antiviral response. However, reduced IFN receptor expression has also been linked to limited responsiveness of human cancers to interferon therapies.^47–49^ As expected, the resulting LLC1-IFNAR1^−/−^ cells were insensitive to interferon-mediated antiviral protection and highly susceptible to VSV-GP infection in vitro. Rendering the cells type 1 IFN-insensitive also translated to a significantly enhanced treatment response in vivo in this otherwise VSV-GP-resistant tumour model. A similar dichotomy in VSV treatment response was previously described for another murine cancer cell line, CT26. Here, the (random) insertion of a reporter gene (CT26LacZ) resulted in a derivative highly permissive for vaccinia, VSV, and other viruses.^33^ In contrast to our system, no single gene defect explains the reduced antiviral phenotype in CT26LacZ cells but rather the downregulation of a pool of genes, which are crucial for innate antiviral responses.

More importantly, in contrast to LLC1 tumours, CT26 are considered highly immunogenic,^41^ with increased immunogenicity of CT26LacZ cells due to the insertion of a foreign immunodominant antigen (LacZ). An important finding from our study is that despite the VSV-GP treatment-related proinflammatory shift in the tumour microenvironment, the immune system does not appear to mount an effective anti-tumour response. In contrast to the CT26LacZ model, protection of mice with long-term remission after VSV-GP treatment of LLC1-IFNAR1^−/−^ tumours against a tumour re-challenge was minimal. Clinical trials have demonstrated that T cell infiltration and PD-L1 upregulation correlate with an increased response probability to immunotherapy.^50,51^ Here, we demonstrate that the viral targeting and lytic processing of LLC1-IFNAR1^−/−^ tumours results in a strong upregulation of immune activation markers such as CD8^+^ T cells and PD-L1 expression. Moreover, immune-focused transcriptome analysis revealed a significant induction of gene signatures for both innate and adaptive immune responses. Yet, the treatment efficacy of VSV-GP on LLC1-IFNAR1^−/−^ tumours does not diminish in the absence of a functional T cell response. When analysing the specificity of CD8^+^ T cells, we monitored a sizeable antiviral T cell response yet failed to detect a response against multiple epitopes of survivin, an LLC-associated antigen.^35^ Even more so, up to 50% of the tumour infiltrating lymphocytes were reactive to a single immune-dominant viral epitope. Compared to TILs, the frequency of antiviral T cells among splenocytes was significantly lower, highlighting the antiviral nature of the strong intratumoural immune activation. It has been well recognised that the immune response triggered by an oncolytic virus can exert effects in both directions: anti-tumour and anti-virus.^2,21^ The dominant trajectory of this response depends on a number of factors, such as the immunogenicity of the virus, the (neo-) antigenic composition and antigen presentation machinery of the tumour cell, the release of soluble danger signals and cytokines, and the tumour microenvironment.

As for the immunogenicity of VSV variants, it is well established that VSV induces strong CD8^+^ T cell responses against virally encoded as well as tumour-related antigens in tumour models.^9^ This immune activation is essential for the VSV treatment effect in certain models, such as B16, as evidenced by therapy failure after CD8^+^ or NK cell depletion.^52^ Interestingly, the same authors report that enhancing effector T cell activity by Treg depletion can actually reduce the anti-tumour response by unleashing forces for faster virus clearance, highlighting the delicate balance between antiviral and anti-tumour immunity. Our finding that absence of CD8^+^ T cells does not decrease the VSV-GP treatment effect on LLC1-IFNAR1^−/−^ tumours runs opposite to the dynamic in B16 tumours. Yet, similar mechanisms might be involved—the difference being that the immune balance is shifted toward antiviral rather than anti-tumour CD8^+^ T-cell activation in the LLC1-IFNAR1^−/−^ model.

The LLC1 tumour has been well established as a poorly immunogenic tumour with downregulated MHC presentation and an immunosuppressive mMDSC-rich tumour microenvironment.^41,53^ Cancer vaccine approaches against survivin have been described,^36^ though due to its self-antigen nature, responses are of low affinity. Other cancer vaccine studies largely reverted to the model antigen chicken ovalbumin (OVA) expressing LLC-OVA tumour cells.^54^ It is conceivable that the lack of immune-dominant antigens in LLC1 tumours further drives the shift of the VSV-GP treatment-induced CD8^+^ T cell response towards a predominant antiviral rather than anti-tumour immunity.

LLC1 tumours showed a downregulation of inflammatory cytokines such as IL-12, IL-5, IL-10 and TNF-α after a cell-based Reovirus vaccine treatment.^55^ In contrast, we detected a significant induction of predominantly inflammatory cytokines IFN-γ, IL-17a, IL-22, MP-1α, RANTES and IP10. This is in line with reports of VSV treatment on B16 tumours with rapid induction of cytokines with anti-tumour activity.^25^ However, in our setting with LLC1-IFNAR1^−/−^ tumours these proinflammatory signals are likely facilitators of the dominating antiviral immune response.

That the treatment effect of VSV-GP on LLC1-IFNAR1^−/−^ tumours is predominantly due to the direct oncolytic activity is further supported by the correlation in magnitude and duration between tumour response and intratumoural virus activity, reported via bioluminescence imaging. In non-responsive LLC1 wt tumours, virus activity could only be detected for the first 24 h post injection of VSV-GP, followed by a sharp drop. Of note, the continued bioluminescence signal in the responsive LLC1-IFNAR1^−/−^ tumours was increased in athymic nude mice compared to C57BL/6J. Although the decreased magnitude of luciferase signal in immune-competent black mice compared to athymic nude mice could in principle be partially explained by differences in skin absorption,^56^ the kinetic of the luciferase signal was profoundly prolonged in athymic nude mice. This further supports the view that antiviral T cells can actively curb VSV-GP virotherapy.

The bioluminescence approach also revealed that VSV-GP can travel via secondary viremia to distant non-injected tumour sites in a syngeneic tumour model with intact immune surveillance. Previously, tumour-to-tumour spread has preclinically been shown predominantly in the xenograft setting using immune-compromised hosts.^57^ In syngeneic hosts, the systemic effect on non-injected tumour lesions has predominantly been described as and attributed to immune-mediated anamnestic effects.^26,58,59^

For virotherapy settings in cases with induction of strong anti-tumour immunity, continued viral replication and spread is often found to be dispensable.^25,60,61^ Naturally, the therapeutic effect in these immune-driven therapeutic responses is often diminished in athymic mice, even though the virus replicates to higher levels.^62^ A treatment effect that heavily depends on the lytic interaction between virus and tumour is rather the exception in syngeneic tumour models. In our setting, we see a complete abrogation of both efficacy as well as the proinflammatory intratumoural cytokine activation when we apply replication-incompetent VSV-ΔG-GP directly into the tumour, further highlighting the dominance of the lytic effect. We previously demonstrated the strong oncolytic potential of VSV-GP in immune-deficient xenograft models using a number of different human cancer models^14–16^ and show here, for the first time, a strong lysis-dominant treatment effect in an immune-competent host. This disconnect between the clearly oncolysis-driven therapeutic effect and the apparently non-contributing immune activation may be relevant for tumours with a very limited repertoire of tumour-associated antigens in which immune-activating interventions may show limited promise. We are aware of a number of model-related limitations and future studies will have to corroborate the relevance of the proposed shift from anti-tumour to antiviral activities. These could include for example testing other tumour derivations such as the well-studied B16 with IFNAR1^−/−^ knockout or the use of oncolytic viruses other than VSV-GP. Together, our results suggest that successful oncolysis does not necessarily induce anti-tumour immunity in select tumour models, despite robust immune activation. An impaired antiviral condition of the target tumour cells provides the permissive environment for this extended lytic activity and this study might have ramifications for rare instances of tumours responding to virotherapy with a lytic pattern and no significant anti-tumour immunity.

## Supplementary information

Supplementary Material

## Data Availability

All pertinent data to support this study are included in the manuscript and supplementary files. Further data supporting the findings are available upon request.

## References

[1] Rehman H, Silk AW, Kane MP, Kaufman HL (2016). Into the clinic: Talimogene laherparepvec (T-VEC), a first-in-class intratumoral oncolytic viral therapy. J. Immunother. Cancer.

[2] Bommareddy PK, Shettigar M, Kaufman HL (2018). Integrating oncolytic viruses in combination cancer immunotherapy. Nat. Rev. Immunol..

[3] Ribas A, Dummer R, Puzanov I, VanderWalde A, Andtbacka RHI, Michielin O (2017). Oncolytic virotherapy promotes intratumoral T cell infiltration and improves Anti-PD-1 immunotherapy. Cell.

[4] Kaufman HL, Kohlhapp FJ, Zloza A (2015). Oncolytic viruses: a new class of immunotherapy drugs. Nat. Rev. Drug Discov..

[5] Koks CA, Garg AD, Ehrhardt M, Riva M, Vandenberk L, Boon L (2015). Newcastle disease virotherapy induces long-term survival and tumor-specific immune memory in orthotopic glioma through the induction of immunogenic cell death. Int. J. Cancer..

[6] Yin J, Markert JM, Leavenworth JW (2017). Modulation of the intratumoral immune landscape by oncolytic herpes simplex virus virotherapy. Front. Oncol..

[7] Hastie E, Grdzelishvili VZ (2012). Vesicular stomatitis virus as a flexible platform for oncolytic virotherapy against cancer. J Gen. Virol..

[8] Stojdl DF, Lichty B, Knowles S, Marius R, Atkins H, Sonenberg N (2000). Exploiting tumor-specific defects in the interferon pathway with a previously unknown oncolytic virus. Nat. Med..

[9] Melzer MK, Lopez-Martinez A, Altomonte J (2017). Oncolytic vesicular stomatitis virus as a viro-immunotherapy: defeating cancer with a “hammer” and “anvil”. Biomedicines..

[10] Lyles, D. S., Rupprecht, C. E. Rhabdoviridae. in *Fields virology* 5th edn. (eds. Knipe, D. M. HP, Griffin, D. E., Lamb, R. A., Straus, S. E., Martin, M. A., Roizman, B.). (Wolters Kluwer/Lippincott Williams & Wilkins, Philadelphia, 2007).

[11] Bishnoi S, Tiwari R, Gupta S, Byrareddy SN, Nayak D (2018). Oncotargeting by vesicular stomatitis virus (VSV): advances in cancer therapy. Viruses.

[12] Critchley-Thorne RJ, Simons DL, Yan N, Miyahira AK, Dirbas FM, Johnson DL (2009). Impaired interferon signaling is a common immune defect in human cancer. Proc. Natl Acad. Sci. USA.

[13] Katsoulidis E, Kaur S, Platanias LC (2010). Deregulation of interferon signaling in malignant cells. Pharmaceuticals.

[14] Dold C, Rodriguez Urbiola C, Wollmann G, Egerer L, Muik A, Bellmann L (2016). Application of interferon modulators to overcome partial resistance of human ovarian cancers to VSV-GP oncolytic viral therapy. Mol. Ther. Oncolytics..

[15] Muik A, Stubbert LJ, Jahedi RZ, Geibeta Y, Kimpel J, Dold C (2014). Re-engineering vesicular stomatitis virus to abrogate neurotoxicity, circumvent humoral immunity, and enhance oncolytic potency. Cancer Res..

[16] Urbiola Carles, Santer Frédéric R., Petersson Monika, van der Pluijm Gabri, Horninger Wolfgang, Erlmann Patrik, Wollmann Guido, Kimpel Janine, Culig Zoran, von Laer Dorothee (2018). Oncolytic activity of the rhabdovirus VSV-GP against prostate cancer. International Journal of Cancer.

[17] Kimpel J, Urbiola C, Koske I, Tober R, Banki Z, Wollmann G (2018). The oncolytic virus VSV-GP is effective against malignant melanoma. Viruses..

[18] Tober R, Banki Z, Egerer L, Muik A, Behmuller S, Kreppel F (2014). VSV-GP: a potent viral vaccine vector that boosts the immune response upon repeated applications. J. Virol..

[19] Russell SJ, Federspiel MJ, Peng KW, Tong C, Dingli D, Morice WG (2014). Remission of disseminated cancer after systemic oncolytic virotherapy. Mayo Clin. Proc..

[20] Marelli G, Howells A, Lemoine NR, Wang Y (2018). Oncolytic viral therapy and the immune system: a double-edged sword against cancer. Front. Immunol..

[21] Prestwich RJ, Errington F, Diaz RM, Pandha HS, Harrington KJ, Melcher AA (2009). The case of oncolytic viruses versus the immune system: waiting on the judgment of Solomon. Hum. Gene Ther..

[22] Speranza MC, Kasai K, Lawler SE (2016). Preclinical mouse models for analysis of the therapeutic potential of engineered oncolytic herpes viruses. ILAR J..

[23] Ungerechts G, Springfeld C, Frenzke ME, Lampe J, Parker WB, Sorscher EJ (2007). An immunocompetent murine model for oncolysis with an armed and targeted measles virus. Mol. Ther..

[24] Falls T, Roy DG, Bell JC, Bourgeois-Daigneault MC (2016). Murine tumor models for oncolytic rhabdo-virotherapy. ILAR J..

[25] Galivo F, Diaz RM, Wongthida P, Thompson J, Kottke T, Barber G (2010). Single-cycle viral gene expression, rather than progressive replication and oncolysis, is required for VSV therapy of B16 melanoma. Gene Ther..

[26] Patel MR, Jacobson BA, Ji Y, Drees J, Tang S, Xiong K (2015). Vesicular stomatitis virus expressing interferon-beta is oncolytic and promotes antitumor immune responses in a syngeneic murine model of non-small cell lung cancer. Oncotarget.

[27] Durham NM, Mulgrew K, McGlinchey K, Monks NR, Ji H, Herbst R (2017). Oncolytic VSV primes differential responses to immuno-oncology therapy. Mol. Ther..

[28] Geiss GK, Bumgarner RE, Birditt B, Dahl T, Dowidar N, Dunaway DL (2008). Direct multiplexed measurement of gene expression with color-coded probe pairs. Nat. Biotechnol..

[29] Kulkarni, M. M. Digital multiplexed gene expression analysis using the NanoString nCounter system. *Curr. Protoc. Mol. Biol*. **94**, 25B.10.1–25B.10.17 (2011). 10.1002/0471142727.mb25b10s94 21472696

[30] Page RD (1996). TreeView: an application to display phylogenetic trees on personal computers. Comput. Appl. Biosci..

[31] Stojdl DF, Lichty BD, tenOever BR, Paterson JM, Power AT, Knowles S (2003). VSV strains with defects in their ability to shutdown innate immunity are potent systemic anti-cancer agents. Cancer Cell..

[32] Noser JA, Mael AA, Sakuma R, Ohmine S, Marcato P, Lee PW (2007). The RAS/Raf1/MEK/ERK signaling pathway facilitates VSV-mediated oncolysis: implication for the defective interferon response in cancer cells. Mol. Ther..

[33] Ruotsalainen JJ, Kaikkonen MU, Niittykoski M, Martikainen MW, Lemay CG, Cox J (2015). Clonal variation in interferon response determines the outcome of oncolytic virotherapy in mouse CT26 colon carcinoma model. Gene Ther..

[34] Zhang L, Steele MB, Jenks N, Grell J, Behrens M, Nace R (2016). Robust oncolytic virotherapy induces tumor lysis syndrome and associated toxicities in the MPC-11 plasmacytoma model. Mol Ther..

[35] Huang TT, Lan YW, Chen CM, Ko YF, Ojcius DM, Martel J (2019). Antrodia cinnamomea induces anti-tumor activity by inhibiting the STAT3 signaling pathway in lung cancer cells. Sci. Rep..

[36] Srivastava AK, Sharma RK, Yolcu ES, Ulker V, MacLeod K, Dinc G (2012). Prime-boost vaccination with SA-4-1BBL costimulatory molecule and survivin eradicates lung carcinoma in CD8+ T and NK cell dependent manner. PLoS One..

[37] Hofmann UB, Voigt H, Andersen MH, Straten PT, Becker JC, Eggert AO (2009). Identification and characterization of survivin-derived H-2Kb-restricted CTL epitopes. Eur. J. Immunol..

[38] Lladser A, Ljungberg K, Tufvesson H, Tazzari M, Roos AK, Quest AF (2010). Intradermal DNA electroporation induces survivin-specific CTLs, suppresses angiogenesis and confers protection against mouse melanoma. Cancer Immunol. Immunother..

[39] Van Bleek GM, Nathenson SG (1990). Isolation of an endogenously processed immunodominant viral peptide from the class I H-2Kb molecule. Nature..

[40] Koske, I., Rossler, A., Pipperger, L., Petersson, M., Barnstorf, I., Kimpel, J., et al. Oncolytic virotherapy enhances the efficacy of a cancer vaccine by modulating the tumor microenvironment. *Int. J. Cancer* **145**, 1958–1969 (2019). 10.1002/ijc.32325 PMC6767478 30972741

[41] Mosely SI, Prime JE, Sainson RC, Koopmann JO, Wang DY, Greenawalt DM (2017). Rational selection of syngeneic preclinical tumor models for immunotherapeutic drug discovery.. Cancer Immunol. Res..

[42] Ilkow CS, Swift SL, Bell JC, Diallo JS (2014). From scourge to cure: tumour-selective viral pathogenesis as a new strategy against cancer. PLoS Pathog..

[43] Krishnamurthy S, Takimoto T, Scroggs RA, Portner A (2006). Differentially regulated interferon response determines the outcome of newcastle disease virus infection in normal and tumor cell lines. J. Virol..

[44] Berchtold S, Lampe J, Weiland T, Smirnow I, Schleicher S, Handgretinger R (2013). Innate immune defense defines susceptibility of sarcoma cells to measles vaccine virus-based oncolysis. J. Virol..

[45] Wollmann G, Robek MD, van den Pol AN (2007). Variable deficiencies in the interferon response enhance susceptibility to vesicular stomatitis virus oncolytic actions in glioblastoma cells but not in normal human glial cells. J. Virol..

[46] Murphy AM, Besmer DM, Moerdyk-Schauwecker M, Moestl N, Ornelles DA, Mukherjee P (2012). Vesicular stomatitis virus as an oncolytic agent against pancreatic ductal adenocarcinoma. J. Virol..

[47] Booy S, van Eijck CH, Dogan F, van Koetsveld PM, Hofland LJ (2014). Influence of type-I Interferon receptor expression level on the response to type-I Interferons in human pancreatic cancer cells. J. Cell. Mol. Med..

[48] Wagner TC, Velichko S, Chesney SK, Biroc S, Harde D, Vogel D (2004). Interferon receptor expression regulates the antiproliferative effects of interferons on cancer cells and solid tumors. Int. J. Cancer.

[49] Saidi RF, Remine SG, Jacobs MJ (2007). Interferon receptor alpha/beta is associated with improved survival after adjuvant therapy in resected pancreatic cancer. HPB..

[50] Herbst RS, Soria JC, Kowanetz M, Fine GD, Hamid O, Gordon MS (2014). Predictive correlates of response to the anti-PD-L1 antibody MPDL3280A in cancer patients. Nature.

[51] Gibney GT, Weiner LM, Atkins MB (2016). Predictive biomarkers for checkpoint inhibitor-based immunotherapy. Lancet Oncol..

[52] Diaz RM, Galivo F, Kottke T, Wongthida P, Qiao J, Thompson J (2007). Oncolytic immunovirotherapy for melanoma using vesicular stomatitis virus. Cancer Res..

[53] Lechner MG, Karimi SS, Barry-Holson K, Angell TE, Murphy KA, Church CH (2013). Immunogenicity of murine solid tumor models as a defining feature of in vivo behavior and response to immunotherapy. J. Immunother..

[54] Yokouchi H, Chamoto K, Wakita D, Yamazaki K, Shirato H, Takeshima T (2007). Combination tumor immunotherapy with radiotherapy and Th1 cell therapy against murine lung carcinoma. Clin. Exp. Metastasis.

[55] Campion CA, Soden D, Forde PF (2016). Antitumour responses induced by a cell-based Reovirus vaccine in murine lung and melanoma models. BMC Cancer.

[56] Curtis A, Calabro K, Galarneau JR, Bigio IJ, Krucker T (2011). Temporal variations of skin pigmentation in C57BL/6 mice affect optical bioluminescence quantitation. Mol. Imaging Biol..

[57] Choi AH, O’Leary MP, Chaurasiya S, Lu J, Kim SI, Fong Y (2018). Novel chimeric parapoxvirus CF189 as an oncolytic immunotherapy in triple-negative breast cancer. Surgery.

[58] Zamarin D, Holmgaard RB, Subudhi SK, Park JS, Mansour M, Palese P (2014). Localized oncolytic virotherapy overcomes systemic tumor resistance to immune checkpoint blockade immunotherapy. Sci. Transl. Med..

[59] Kaufman HL, Amatruda T, Reid T, Gonzalez R, Glaspy J, Whitman E (2016). Systemic versus local responses in melanoma patients treated with talimogene laherparepvec from a multi-institutional phase II study. J. Immunother. Cancer..

[60] Lemay CG, Rintoul JL, Kus A, Paterson JM, Garcia V, Falls TJ (2012). Harnessing oncolytic virus-mediated antitumor immunity in an infected cell vaccine. Mol. Ther..

[61] Oseledchyk A, Ricca JM, Gigoux M, Ko B, Redelman-Sidi G, Walther T (2018). Lysis-independent potentiation of immune checkpoint blockade by oncolytic virus. Oncotarget..

[62] Leddon JL, Chen CY, Currier MA, Wang PY, Jung FA, Denton NL (2015). Oncolytic HSV virotherapy in murine sarcomas differentially triggers an antitumor T-cell response in the absence of virus permissivity. Mol. Ther. Oncolytics.

