# Supplementary material for: The lytic activity of VSV-GP treatment dominates the therapeutic effects in a syngeneic model of lung cancer
Source: Br J Cancer. 2019 Sep 18;121(8):647–58. doi: 10.1038/s41416-019-0574-7 (PMC6889376; doi:10.1038/s41416-019-0574-7)
Supplement: Supplementary file 1 — Supplementary Material [file 41416_2019_574_MOESM1_ESM.pdf]

SUPPLEMENTARY MATERIAL

Supplemental Methods S1

Animal housing. Animals were housed in a non-SPF, BL2 infectious agent facility. C57BL/6J mice were kept in individually ventilated cages (IVC), Euro Standard Type IIL in groups of 5, NMRI nu/nu mice were housed in groups of 10 in Euro Standard Type III cages with filter tops (both Techniplast, Germany). Temperature in animal facilities was 20-24 °C for C57BL/6J and 22-26 °C for NMRI nu/nu, humidity was 55 ± 10%. Light cycle was 12 hours. Housing was enriched by nesting material, plastic houses as well as wooden sticks; food and water were available ad libitum. Well-being of the animals was monitored daily.

Experimental procedures. Treatment was applied during daytime in a separate procedure room in a BSL2 safety cabinet. Tumor grafting, i.t. virus injection and bioimaging were conducted under isoflurane anesthesia using the XGI-8 Gas Anesthesia System (2-3 % isoflurane, 2-2.5 l/min, mixed with O_2; Caliper Life Sciences). For terminal blood collection, animals were anesthetized by ketamin/xylazin (i.p., 80-100 mg/kg and 5-10 mg/kg). Animals were euthanized by CO_2 asphyxiation and cervical dislocation or via short-term isoflurane anesthesia followed by cervical dislocation. Humane endpoint criteria were defined as tumor size of > 0.8 and < 1.0 cm^3, tumor ulceration or bleeding, weight loss > 20%, poor general condition or neurological abnormalities.

Treatment group allocation. Animals were allocated to treatment groups at the day before treatment start when median tumor volume reached a size of 0.05 to 0.07cm^3. Treatment groups were randomized with comparable median tumor size in each group. The smallest and largest tumors were evenly distributed. Group variance was comparable between groups. For tumor treatment, PBS group was first, followed by virus treatment groups in increasing doses. Within a treatment group, mice were taken from the housing cage for treatment intervention at random. For tumor measurements, order within one group was random. Order of groups was as with treatment listed above. Depending on the experimental read-out, treatment groups consisted of 3-10 mice. Based on long-term experience with VSV-GP as an oncolytic agent *in vivo*, 8-10 mice

per group are required for efficacy studies in tumor bearing animals on most models. For immunohistochemical or NanoString analysis, multiplex ELISA or flow cytometry analysis of tumor infiltrating immune cells, 3-6 samples per treatment group were found to be required for consistency.

In vivo studies were performed as a single run including all corresponding controls; treatment effect was replicated in numerous studies with additional read-outs (e.g. dose response, bioluminescence). To reduce animal numbers, methods were established in a way that enabled different readouts from a single animal (histological analysis, transcriptome and cytokine analysis from a single tumor). Using bioimaging to longitudinally monitor virus activity in tumors reduced the need to sacrifice the host.

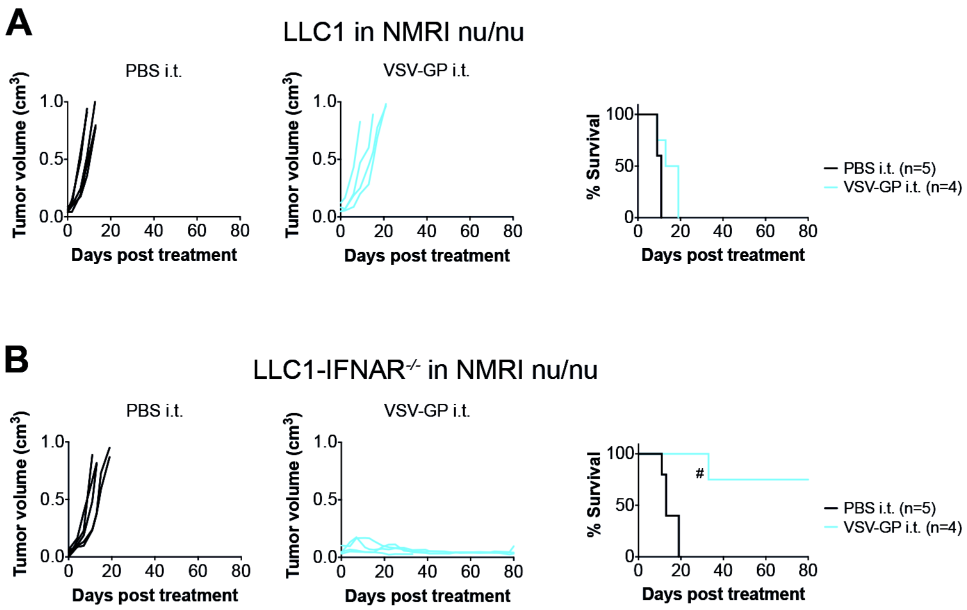

Fig. S1: Efficacy of VSV-GP treatment on LLC1 or LLC1-IFNAR1$^{-/-}$ tumors in immune-incompetent mice.

Tumors were implanted in T-cell-deficient NMRI-nu/nu mice by subcutaneously injecting $5x10^5$ LLC1 wt **(A)** or $1x10^6$ IFNAR1$^{-/-}$ cells **(B)** into the right flank. Intratumoral treatment with VSV-GP at a dosis of 10^8 $TCID_{50}$ was initiated when tumors reached a size of 0.05-0.07cm^3. Individual tumor volume graphs and Kaplan–Meier survival curves are shown. # indicates a non-tumor related event.

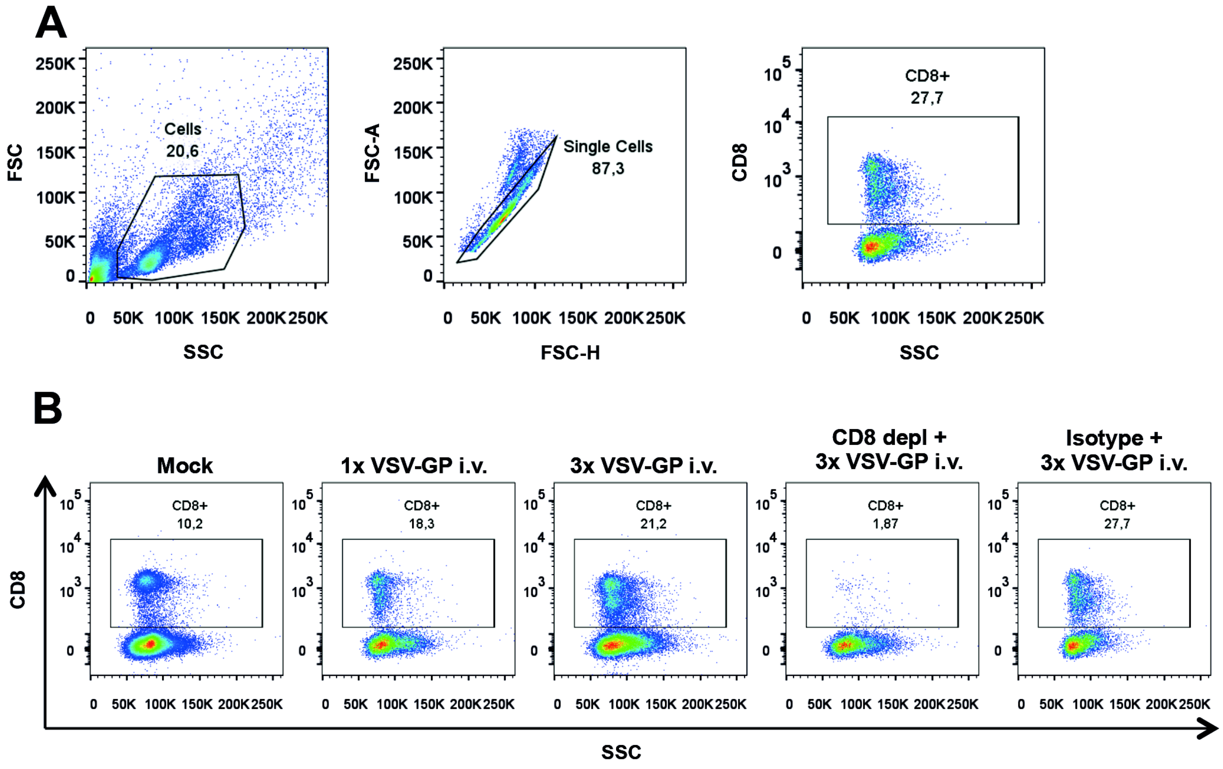

Fig. S2: CD8$^+$ T-cell depletion – gating strategy and depletion validation.

Depletion of CD8 T-cells was confirmed by blood collection from all experimental animals. Cells were gated based on their forward and side scatter characteristics followed by exclusion of doublets. Percentage of CD8 positive T-cells was determined using a monoclonal anti-mouse CD8a antibody**.** Respective pseudocolor density plots from each treatment group are shown.

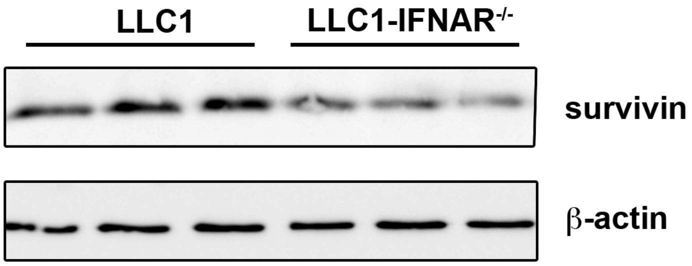

Fig. S3: Expression of survivin by parental and IFNAR1$^{-/-}$ LLC1 cells.

Lysates of monolayer LLC1 wt or IFNAR1$^{-/-}$ cells were prepared and analyzed for survivin expression by Western Blot; β-actin was used as loading control. Cells were lysed in ice-cold 1x RIPA buffer containing 1mM PMSF for 30 min and centrifuged at 15,000g for 10 min to remove cell debris. Protein separation was performed by sodium dodecyl sulfate (SDS)-polyacrylamide gel electrophoresis (PAGE) under standard reducing conditions on a 10% polyacrylamide gel. Proteins were transferred by electrophoresis to a 0.45 μm nitrocellulose membrane (Whatman, Dessel, Germany). Membranes were blocked with TBST-milk (TBS containing 0.1% Tween-20 and 5% milk (AppliChem, Darmstadt, Germany)) and stained overnight at 4°C with primary antibodies. Detection was done using horseradish peroxidase (HRP) specific secondary antibodies. The following antibodies were used: anti-survivin from rabbit (Thermo Fisher Scientific), anti-β-actin from mouse (clone AC-74, Sigma-Aldrich), HRP-conjugated anti-rabbit from goat and HRP-conjugated anti-mouse from goat (Jackson ImmunoResearch).

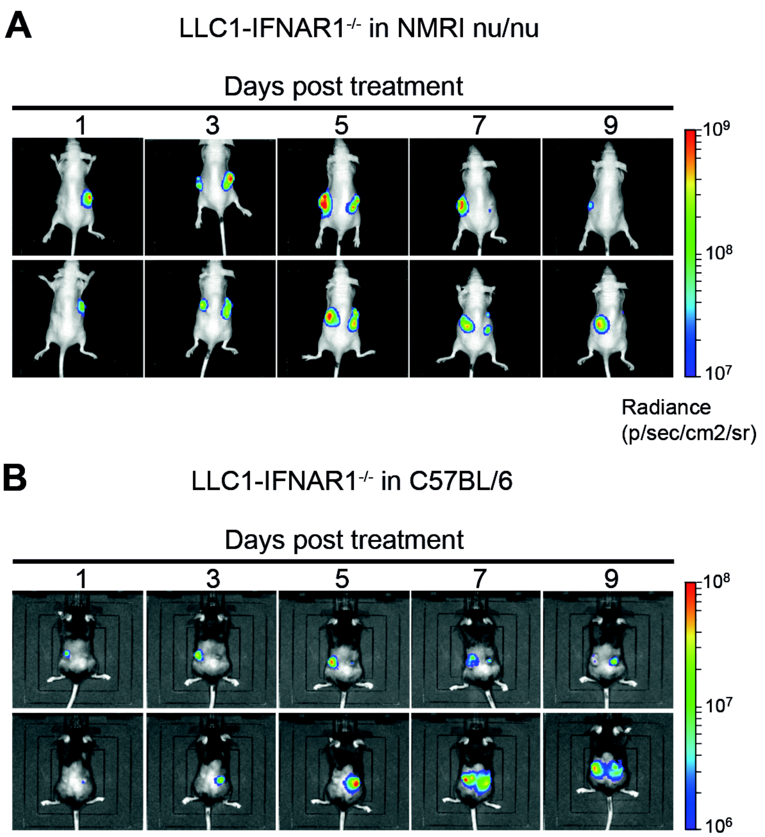

Fig. S4: Tumor-to-tumor spread of VSV-GP.

Bilateral subcutaneous LLC1-IFNAR1$^{-/-}$ tumors were grown in either T-cell deficient athymic NMRI-nu/nu mice **(A)** or syngeneic C57BL/6J mice **(B)** by injecting $3x10^5$ LLC1-IFNAR1$^{-/-}$ cells subcutaneously into both flanks. The smaller of the two tumors was treated at size of $0.05cm^3$ with an intratumoral injection of 10^8 $TCID_{50}$ of VSV-GP-Luciferase and subjected to bioluminescence imaging every second day post treatment. BLI signal can be detected at the non-injected site within 3 and 7 days for NMRI-nu/nu and C57BL/6J hosts, respectively. The panel depicts representative pictures of a group size of 5 animals each. Color scale displays luminescence as photons/second/cm^2/steradian (p/s/cm^2/sr).

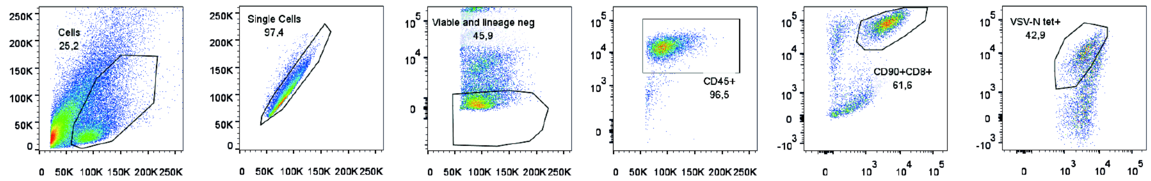

Fig. S5: Gating strategy for VSV-N-specific CD8^{+} T cells among tumor infiltrating lymphocytes (TILs).

Cells were gated based on their forward and side scatter characteristics followed by exclusion of doublets, non-viable cells, and cells expressing lineage markers for monocytes (CD14^{+}), myeloid cells (CD11b^{+}) and B cells (CD19^{+}). Leukocytes were identified by CD45^{+} surface staining and CD8^{+} lymphocytes were further defined by high expression of CD90 and CD8 molecules. Among CD90^{+}CD8^{+} T cells, VSV-NP tetramer positive cells were defined as VSV-GP specific CD8^{+} T cells.

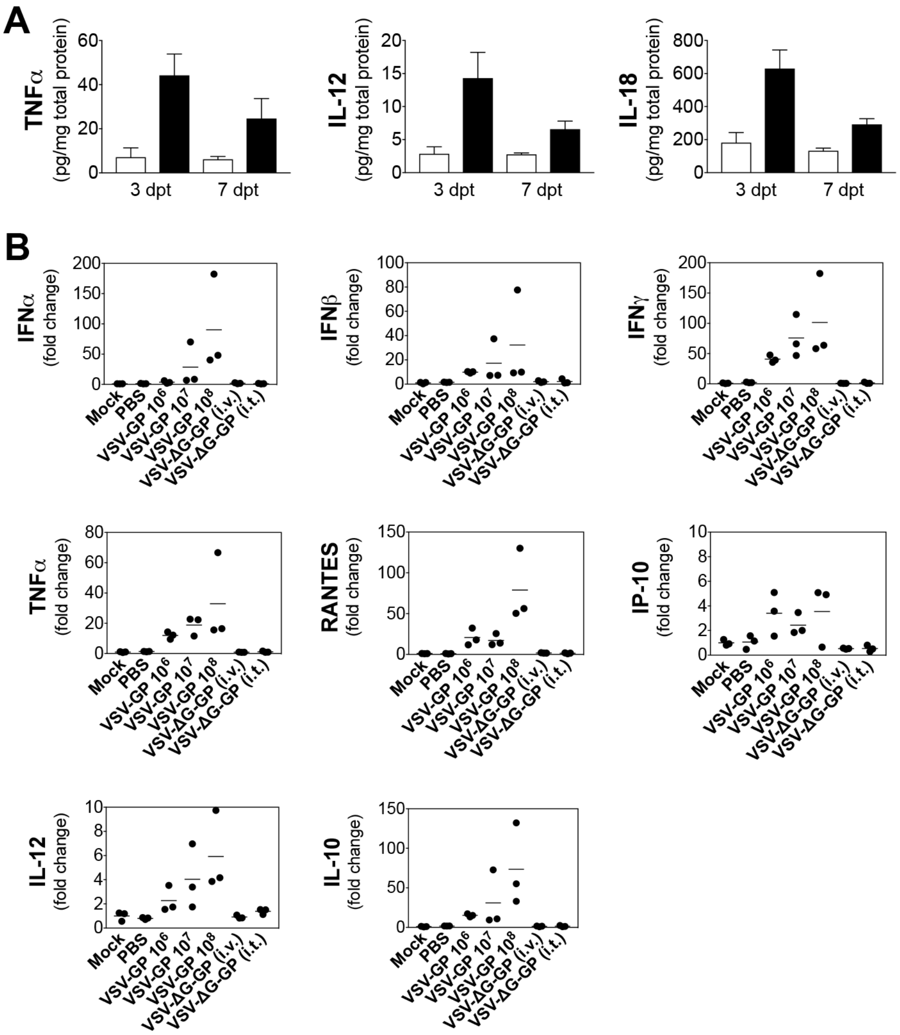

Fig. S6: VSV-GP treatment induces cytokines in LLC1-IFNAR1$^{-/-}$ tumors.

A) LLC1-IFNAR1$^{-/-}$ tumors were treated with 10^8 $TCID_{50}$ VSV-GP (i.v.) and harvested at indicated time points, processed and analyzed using multiplex cytokine assays. 3 and 7 days post infection, upregulation of TNFα, IL-12 and IL-18 was observed, although not significant. Data presented as mean ± SEM (n=3). **B)** Tumors treated with 10^6 to 10^8 $TCID_{50}$ VSV-GP i.v. or replication-incompetent VSV-ΔG-GP i.v./i.t. were harvested after 7 days, processed and analyzed. Intratumoral cytokines showed strong dose-dependency of VSV-GP treatment. Replication-deficient VSV-GP variant VSV-ΔG-GP did not induce intratumoral cytokine upregulation. Data presented as mean (n=3).

Table S1: Innate immune signature.

Innate immune signature – Treated vs Mock					
**Probe Label**	**Fold change**	**Probe Label**	**Fold change**	**Probe Label**	**fold change**
Cxcl10-mRNA	7.78124	Syk-mRNA	1.796265	Traf2-mRNA	1.044201
Slamf7-mRNA	7.210004	Cyld-mRNA	1.771535	Clec7a-mRNA	1.025409
Ccl5-mRNA	7.210004	Clec4n-mRNA	1.765406	Irak4-mRNA	1.021508
Isg15-mRNA	5.028053	Tlr2-mRNA	1.758079	Itch-mRNA	1.017762
Ifih1-mRNA	4.756828	Traf3-mRNA	1.745935	Ly96-mRNA	1.011713
Cd180-mRNA	4.563055	Btk-mRNA	1.720705	Cd14-mRNA	1.006021
Irf7-mRNA	4.531536	C1qb-mRNA	1.718322	Rela-mRNA	1.004683
C3-mRNA	4.500234	Nod1-mRNA	1.713564	Tlr4-mRNA	1.004383
Cfb-mRNA	4.40762	Clec4a2-mRNA	1.69937	Map2k1-mRNA	0.992886
Tnfaip3-mRNA	4.084049	Cxcl16-mRNA	1.658639	Mapk3-mRNA	0.990549
Zbp1-mRNA	3.944931	Nfkbia-mRNA	1.613284	Ccl2-mRNA	0.97725
Serping1-mRNA	3.89062	Nod2-mRNA	1.60214	Tollip-mRNA	0.970702
Stat1-mRNA	3.837056	Map3k1-mRNA	1.60103	Bcl2l1-mRNA	0.963262
Ddx58-mRNA	3.5801	Ccr1-mRNA	1.59549	Ifitm2-mRNA	0.939002
Tlr9-mRNA	3.458149	Ifngr1-mRNA	1.516768	Irf3-mRNA	0.932386
Cd74-mRNA	3.24901	Jak3-mRNA	1.50629	Tbk1-mRNA	0.920826
Nlrc5-mRNA	3.160165	Casp8-mRNA	1.501079	Ikbkg-mRNA	0.907519
Cd36-mRNA	3.116658	Csf1-mRNA	1.472227	Map3k5-mRNA	0.898755
Abca1-mRNA	3.07375	Casp1-mRNA	1.45902	Il1rl1-mRNA	0.886382
Abcg1-mRNA	3.031433	Mapkapk2-mRNA	1.451958	Chuk-mRNA	0.87843
Cd55-mRNA	2.989698	Itgam-mRNA	1.438934	Map2k4-mRNA	0.866337
Fcgr1-mRNA	2.789487	Ubc-mRNA	1.428004	Cd1d1-mRNA	0.862741
Isg20-mRNA	2.770219	Nfkb1-mRNA	1.403472	Mapk1-mRNA	0.852044
Il18r1-mRNA	2.675855	Cebpb-mRNA	1.392811	Map3k7-mRNA	0.852044
Irgm2-mRNA	2.566852	Crebbp-mRNA	1.380317	Lcn2-mRNA	0.829895
Pik3cd-mRNA	2.531513	Jak1-mRNA	1.339784	Bid-mRNA	0.815637
Mefv-mRNA	2.394957	Myd88-mRNA	1.298639	Bcl10-mRNA	0.805524
Abl1-mRNA	2.378414	Txnip-mRNA	1.295043	Dusp6-mRNA	0.787854
C1ra-mRNA	2.329467	Il1b-mRNA	1.285206	Creb1-mRNA	0.781328
C1s1-mRNA	2.313376	Clec5a-mRNA	1.278986	Atf1-mRNA	0.760489
Pik3cg-mRNA	2.250117	Lgals3-mRNA	1.251796	Atg12-mRNA	0.758384
Fos-mRNA	2.234574	Cd97-mRNA	1.221793	Clu-mRNA	0.75106
Tlr8-mRNA	2.234574	Tank-mRNA	1.212513	Il1rap-mRNA	0.734584
Cybb-mRNA	2.188587	Ep300-mRNA	1.204972	Tab1-mRNA	0.72749
App-mRNA	2.17347	Itga5-mRNA	1.155887	Hmgb1-mRNA	0.725979
Xcl1-mRNA	2.158456	Ripk2-mRNA	1.146312	Cxcr2-mRNA	0.723467
Tlr1-mRNA	2.143547	Ikbkb-mRNA	1.145518	Atf2-mRNA	0.696406
Csf1r-mRNA	2.12874	Irak2-mRNA	1.137605	Il1r1-mRNA	0.675955
Ctss-mRNA	2.099433	Nfkb2-mRNA	1.118837	Ifitm1-mRNA	0.653836
Cxcl9-mRNA	2.099433	Mapk14-mRNA	1.106497	Irak1-mRNA	0.65293
Hck-mRNA	2.099433	Traf6-mRNA	1.077733	Mif-mRNA	0.624598
Bst2-mRNA	2.07053	Tmem173-mRNA	1.075494	Il18rap-mRNA	0.595016
Jak2-mRNA	2.042024	Colec12-mRNA	1.066438	Dusp4-mRNA	0.592957
Ly86-mRNA	2.027919	Axl-mRNA	1.061718	Il34-mRNA	0.50698
Cfp-mRNA	1.954772	Map2k2-mRNA	1.0601	Mapk8-mRNA	0.5
C1qa-mRNA	1.85961	Cxcl2-mRNA	1.054018	Masp1-mRNA	0.486327
Ncf4-mRNA	1.823866	Cxcl2-mRNA	1.054018	Sigirr-mRNA	0.460094
Lyn-mRNA	1.796265	Cfh-mRNA	1.04819		

Table S1: Innate immune signature. List of genes linked to innate immune responses that are up- or downregulated in tumors harvested after VSV-GP treatment compared to mock as depicted in Fig. 6D.

Table S2: Adaptive immune signature.

Adaptive immune signature – Treated vs Mock			
**Probe Label**	**Fold change**	**Probe Label**	**Fold change**
Cxcl10-mRNA	7.78124	Itgam-mRNA	1.438934
Ccl5-mRNA	7.210004	Nfkb1-mRNA	1.403472
Irf7-mRNA	4.531536	Il6st-mRNA	1.394744
Stat1-mRNA	3.837056	Ccr7-mRNA	1.372684
Ccrl2-mRNA	3.837056	Ccl7-mRNA	1.363203
Fcer1a-mRNA	3.116658	Cma1-mRNA	1.353786
Thbs1-mRNA	2.8481	Cd80-mRNA	1.320423
Ccl8-mRNA	2.713209	Slc11a1-mRNA	1.312211
Ccl4-mRNA	2.675855	Nos2-mRNA	1.307671
Cxcl14-mRNA	2.639016	Il1b-mRNA	1.285206
Ccl11-mRNA	2.620787	Tnfrsf11a-mRNA	1.260503
Ccr5-mRNA	2.297397	Cd97-mRNA	1.221793
C3ar1-mRNA	2.158456	H2-Q10-mRNA	1.181812
Cxcl9-mRNA	2.099433	Cxcl2-mRNA	1.054018
Cd86-mRNA	2.056228	Cxcl3-mRNA	1.030754
Jak2-mRNA	2.042024	Tlr4-mRNA	1.004383
Ccl3-mRNA	2.042024	Stat6-mRNA	0.980099
Ccl12-mRNA	2.013911	Creb5-mRNA	0.942915
Fpr2-mRNA	1.995845	Irf3-mRNA	0.932386
Cd40-mRNA	1.979313	Hmgb1-mRNA	0.725979
Icam1-mRNA	1.888185	Cxcr2-mRNA	0.723467
Stat3-mRNA	1.704088	Il1r1-mRNA	0.675955
Ifnar1-mRNA	1.649467	Cklf-mRNA	0.573554
Il18-mRNA	1.635804	C1qbp-mRNA	0.533663
Nod2-mRNA	1.60214	Mapk8-mRNA	0.5
Ccr1-mRNA	1.59549	S100a8-mRNA	0.476319
Ccr2-mRNA	1.575708	Cxcl1-mRNA	0.417544
Ifngr1-mRNA	1.516768		

Table S2: Adaptive immune signature. List of genes linked to adaptive immune responses that are up- or downregulated in tumors harvested after VSV-GP treatment compared to mock as depicted in Fig. 6D.
